# Regulation and roles of RNA modifications in aging‐related diseases

**DOI:** 10.1111/acel.13657

**Published:** 2022-06-19

**Authors:** Zeyidan Jiapaer, Dingwen Su, Lingyang Hua, Helge Immo Lehmann, Priyanka Gokulnath, Gururaja Vulugundam, Shannan Song, Lingying Zhang, Ye Gong, Guoping Li

**Affiliations:** ^1^ College of Life Science & Technology Xinjiang University Urumqi China; ^2^ Xinjiang Key laboratory of Biological Resources and Genetic Engineering Urumqi China; ^3^ Friedrich Miescher Laboratory of the Max Planck Society Tübingen Germany; ^4^ Department of Neurosurgery, Huashan Hospital, Shanghai Medical College Fudan University Shanghai China; ^5^ Cardiovascular Research Center Massachusetts General Hospital and Harvard Medical School Boston MA USA; ^6^ Institute of Biochemistry and Cellular Biology National Research Council of Italy Naples Italy

**Keywords:** aging, aging‐related disease, epitranscriptome, RNA modification

## Abstract

With the aging of the global population, accumulating interest is focused on manipulating the fundamental aging‐related signaling pathways to delay the physiological aging process and eventually slow or prevent the appearance or severity of multiple aging‐related diseases. Recently, emerging evidence has shown that RNA modifications, which were historically considered infrastructural features of cellular RNAs, are dynamically regulated across most of the RNA species in cells and thereby critically involved in major biological processes, including cellular senescence and aging. In this review, we summarize the current knowledge about RNA modifications and provide a catalog of RNA modifications on different RNA species, including mRNAs, miRNAs, lncRNA, tRNAs, and rRNAs. Most importantly, we focus on the regulation and roles of these RNA modifications in aging‐related diseases, including neurodegenerative diseases, cardiovascular diseases, cataracts, osteoporosis, and fertility decline. This would be an important step toward a better understanding of fundamental aging mechanisms and thereby facilitating the development of novel diagnostics and therapeutics for aging‐related diseases.

## Graphical abstract

RNA modificaitons in aging‐related diseases

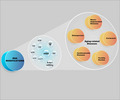

**Abbreviations**
2′‐OMe: 2′‐O‐methylation
3′UTR: 3′ untranslated regions
AD: Alzheimer's disease
ADAR: adenosine deaminases acting on the RNA
ALS: amyotrophic lateral sclerosis
A‐to‐I editing: adenosine to inosine RNA editing
BS‐seq: bisulfite sequencing
CHAPIR: cardiac‐hypertrophy‐associated piRNA
Cm: 2′‐O‐methylcytidine
CTSS: Cathepsin S
DART‐seq: deamination adjacent to RNA modification targets sequencing
DKC1: dyskerin
Ψ: pseudouridine
FTO: alpha‐ketoglutarate‐dependent dioxygenase FTO
LECs: lens epithelium cells
LC: liquid chromatography
m1A: N1‐methyladenosine
m1G: 1‐methyl‐guanosine
m2,2,7G: N2, N2, 7‐trimethylguanosine
m5C: 5‐Methylcytosine
m6A: N6 methylation of adenosine
m6A‐SAC‐seq: m6A selective allyl chemical labeling and sequencing
m6A‐SNPs: m6A‐associated single‐nucleotide polymorphisms
m7G: 7‐methylguanosine
m7G‐MaP‐seq: m7G mutational profiling sequencing
METTL3: methyltransferase‐like 3
MS: mass spectrum
pri‐miRNAs: primary microRNAs
PD: Parkinson's disease
PUS: pseudouridine synthases
Q/R: glutamine/arginine
snoRNAs: small nucleolar RNAs
SNPs: single nucleotide polymorphisms
TAC: transverse aortic constriction
UPR: unfolded protein response
YTHDF1: YTH domain‐containing family protein 1

## 1 INTRODUCTION

Aging is a natural gradually occurring process of progressive decline in an organism's physiological and psychological adaptability to the environment, culminating in its death. Molecular hallmarks of aging include genomic instability, telomere attrition, epigenetic alterations, loss of proteostasis, deregulated nutrient sensing, mitochondrial dysfunction, cellular senescence, stem cell exhaustion, and altered intercellular communication. In humans, aging can be correlated to increased risks of multiple diseases such as neurodegenerative diseases, cardiovascular diseases, osteoporosis, metabolic dysfunction, defective tissue repair and regeneration, decreased regulation of gut microbes, and cataracts (Cui et al., 2017; Lopez‐Otin et al., 2013). Accumulating studies on aging have revealed that aging phenotypes and aging‐related diseases usually result from the complicated interaction between an external environmental stimulus and internal gene expression regulation. Concerning epigenetically regulated gene expression in aging, though most attention has been given to transcriptional alterations, such as DNA methylation patterns, histone modifications, and chromatin remodeling, post‐transcriptional regulators, in particular RNA modifications, have been previously underestimated due to the lack of relevant tools to investigate them (Saul & Kosinsky, 2021). One of the first hints in this direction was speculated by a study analyzing the transcriptomes of young and aging mouse livers, which showed that differentially expressed genes in the aging mice liver were significantly enriched in RNA modification‐related pathways (White et al., 2015).

RNA modifications play a critical role in nearly every aspect of the biological process, ranging from early embryo development until aging (Mendel et al., 2018). The existence of modified RNA bases was firstly discovered via enzymatic digestion and electrophoresis in the early 1960s and 1970s (Lavi et al., 1977). Thin‐layer chromatography, high‐performance liquid chromatography (LC), and mass spectrum (MS) were then utilized to determine and detect RNA nucleobase modification based on the differences in the biophysical and biochemical properties between the modified and unmodified bases such as molecular mass, net charge, polarity, and hydrophobicity (Delatte et al., 2016; Jia et al., 2011; Shen et al., 2019). Although these methods are less quantitative, not high‐throughput, and may miss the specific RNA context for a modification in some cases, they have expanded the world of RNA modifications. Recently, the next‐generation sequencing‐based methods with or without using RNA modification specific antibodies, such as m6A‐seq, m6A‐selective allyl chemical labeling and sequencing (m6A‐SAC‐seq), deamination adjacent to RNA modification targets (DART‐seq), 7‐methylguanosine mutational profiling sequencing (m7G‐MaP‐seq), bisulfite sequencing (BS‐seq), and comparative Nanopore direct RNA sequencing, have been developed to probe the specific RNA modification in the whole transcriptome at single‐base resolutions (Enroth et al., 2019; Helm & Motorin, 2017; Hu et al., 2022; Leger et al., 2021; Li et al., 2017; Meyer, 2019). Owing to the great advent of methods to detect them qualitatively and quantitatively, hundreds of RNA modifications have now been identified in mammalian cells and all known RNA species, including mRNA, miRNA, tRNA, rRNA, lncRNA, and other non‐coding RNAs (Frye et al., 2016), further boosting the epitranscritome studies and suggesting their exigent biological functions. RNA modifications have been reported to critically contribute to nuclear export, translation initiation, transcript stability, splicing, folding, and localization (Roundtree et al., 2017). Along with the discoveries of RNA modifications, some enzymes that are responsible for writing, reading, and erasing these RNA modifications have also been identified (Kumar & Mohapatra, 2021). RNA modifications and corresponding modifying enzymes are gaining increasing attention due to their pivotal roles in numerous human diseases, such as obesity, diabetes, neurodegenerative diseases, multiple types of cancer, and even viral infections (Chatterjee et al., 2021; Zhang et al., 2021; Zhou et al., 2020). Many of these linkages arise from mutations and/or single nucleotide polymorphisms (SNPs) in RNA modification‐related genes and pathways.

The biochemical aspects of RNA modifications have been extensively reviewed elsewhere (Harcourt et al., 2017). Here, we will introduce the biogenesis and molecular functions of the relatively well‐studied RNA modifications in different RNA species. Further, we will focus on the regulation and roles of RNA modifications in aging‐related diseases, including neurodegenerative diseases, cardiovascular diseases, cataracts, osteoporosis, and fertility decline.

## 2 RNA MODIFICATIONS

### 2.1 Modifications on mRNA

**FIGURE 1:**
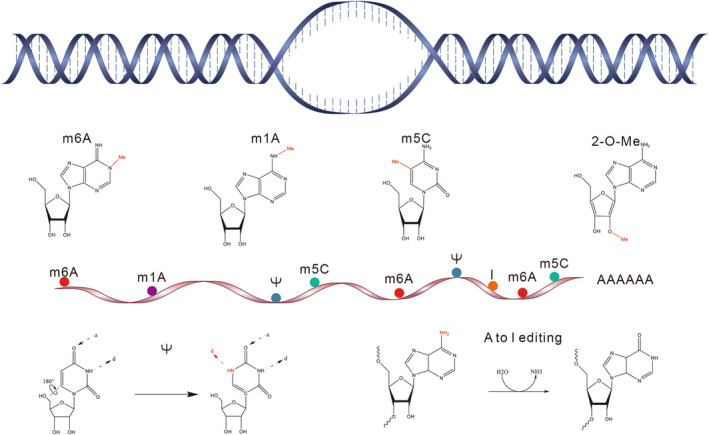
Chemical modifications in mRNA

The recent discovery of reversible mRNA chemical modifications has opened a new era of post‐transcriptional gene regulation in living organisms. Apart from the 5′ cap and 3’poly(A) tail in mature eukaryotic mRNAs, more than 100 distinct chemical modifications have been found to actively regulate mRNA behaviors, including differentially processing, splicing, translation, and decay (Boccaletto et al., 2022). Here, we will only focus on the main modifications that are reported to be critically involved in the regulation of mRNA functions (Figure 1).

#### 2.1.1 N6 methylation of adenosine (m6A)

The modification of m6A was first identified in 1970s (Dubin & Taylor, 1975). With the advances in identifying and quantifying m6A in the transcriptome at single‐base resolution (Hu et al., 2022; Linder et al., 2015), m6A becomes the best‐characterized and most abundant internal RNA modification with about 0.2%–0.6% of adenosines having m6A in mammalian mRNAs (Molinie et al., 2016). The transcriptome‐wide m6A distribution in mice and humans revealed that m6A is enriched in the coding region and 3′ untranslated regions (3’ UTR), with a significant enrichment near the stop codon (Dominissini et al., 2012; Meyer et al., 2012). m6A modification is catalyzed by METTL3‐METTL14 complex and their cofactors, such as METTL16, ZCCHC4, RBM15, ZC3H13, VIRMA, CBLL1, and WTAP (Knuckles & Buhler, 2018; Liu et al., 2014; Warda et al., 2017; Wen et al., 2018; Yue et al., 2018). YTHDF1, YTHDF2, IGF2BP1, IGF2BP2, and IGF2BP3 are characterized as reader proteins that recognize the m6A methylation. Two prominent demethylation enzymes are FTO and ALKBH5. Functionally, m6A is involved in almost every step in the mRNA life cycle, from splicing and processing in the nucleus to translation and decay in the cytoplasm (Zhao et al., 2017). At the cellular level, m6A plays a critical role in cellular identity transition between distinct states during differentiation or stress response via influencing the transcriptome output (Zhao et al., 2017).

#### 2.1.2 N1 methylation of adenosine (m1A)

m1A is the methylation of the N1 position of adenosine which was identified in 1961 in tRNA and rRNA (Dunn, 1961). Recently, its presence in eukaryotic mRNA has been demonstrated and its transcriptome‐wide distribution has also been mapped via high‐throughput methods (Dominissini et al., 2016; Li et al., 2016). m1A is highly enriched around the start codon within the 5’UTR and is preferentially located in highly structured areas (Dominissini et al., 2016; Li et al., 2016). A recent study showed that the presence of m1A blocks reduces RNA base‐pairing and induces local RNA duplex melting (Zhou et al., 2016). m1A has been shown to promote translation (Dominissini et al., 2016; Li et al., 2016), although the detailed molecular mechanism is not clear. The only known methyltransferase catalyzing m1A on mRNA is the TRMT6–TRMT61 complex (Safra et al., 2017). It has been reported that YTHDF2 is not only the reader of m6A but also can bind with low affinity to m1A, which suggests its potential role as an m1A reader in cells (Dai et al., 2018). Moreover, the known erasers of m1A in mRNA are ALKBH1 and ALKBH3 (Aas et al., 2003; Liu et al., 2016).

#### 2.1.3 7‐Methylguanosine (m7G)

m7G is a positively charged RNA modification that is modified by the addition of the 7‐methylguanosine “cap” added to the first transcribed nucleotide, which is necessary for the translation of the majority of mRNAs (Cowling, 2009). RNMT is the first identified cap methyltransferase catalyse (Trotman et al., 2017). The reader and eraser proteins are yet to be identified.

#### 2.1.4 2’O‐methylation (2’‐OMe)

2’‐OMe is one of the classical RNA modifications wherein 2′ hydroxyl (–OH) groups have been added to the ribose. The 2’‐OMe modification was first demonstrated in bacterial mRNA to affect translation efficiency (Hoernes et al., 2016). 2’‐OMe can be added on the N1 (first transcribed nucleotide, m7GpppNmN‐) and also on the N2 (second transcribed nucleotide, m7GpppNmNm‐), respectively. CMTR1 may act as 2’‐O‐methyltransferase that modifies the N1 of the mRNA cap (Belanger et al., 2010). There is a need to develop highly sensitive and quantitative methods to gather more information regarding this modification.

#### 2.1.5 5‐Methylcytosine (m5C)

m5C is the most common modification of mRNA where methylation occurs at the 5th position of cytosine and was first reported in 1975. This modification is similar to DNA methylation m5C except for the ribose. NSUN2 is associated with this mRNA modification. The m5C modification could also be recognized by the mRNA export adaptor protein ALYREFRNA (Bohnsack et al., 2019; Yang et al., 2017), while the exact methyltransferase(s) responsible for m5C modifications in mRNAs is yet to be identified.

#### 2.1.6 Pseudouridine

Pseudouridine, or isomerization of uridine, also known as 5‐ribosyl uracil and Ψ, was first discovered in 1957. This was initially identified as the fifth base in RNA due to its high abundance in cellular RNA. This modification in mRNA is partially attributed to several tRNA and rRNA pseudouridine synthases (PUS) conserved across eukaryotes (Eyler et al., 2019). Further biochemical research and understanding of Ψ in mRNAs are required. Due to its low abundance in mRNA, this modification has not been studied properly until recent technological advances such as the establishment of PseudoU‐seq. This results from the post‐transcriptional isomerization reaction of uridine (1‐ribosyl uracil), which makes pseudouridine carry distinct chemical and biophysical properties compared with uridine. This rigidifies both single‐stranded and duplex RNA locally, and thus restricts their flexibility. Expectedly, pseudouridine can affect the secondary structure of mRNA. This modification can have a substantial impact on the translation process and the outcome of translation especially when it occurs in the stop codons or nonsense codons, given that all stop codons start with U at the first base. Ψ‐containing codons have been shown to be able to modestly affect the ribosomes, incorporating certain amino acids and Ψ‐containing stop codons that have been observed to direct the nonsense suppression of translation termination (Eyler et al., 2019; Fernandez et al., 2013; Karijolich & Yu, 2011).

#### 2.1.7 Adenosine to inosine RNA editing (A‐to‐I editing)

Like RNA modification, A‐to‐I editing is a common event in the transcriptome. This conversion is catalyzed by adenosine deaminases acting on the RNA (ADAR) family of enzymes on both intermolecular and intramolecular double‐stranded RNAs longer than 20 bp. Mammals have 3 ADAR enzymes, ADAR1 and ADAR2 being catalytically active while ADAR3 lacks catalytic activity. Considering both coding and non‐coding transcripts, tens of thousands of A‐to‐I editing sites have been identified in mice and millions have been identified in humans. A‐to‐I editing levels vary across transcripts, tissues, and throughout development ranging from 1 to 100 percent at any given site (Porath et al., 2017; Tan et al., 2017).

Compared with adenosine, inosine has distinct thermodynamic base‐pairing properties that could lead to possible alterations in the secondary structure and encoded information. Inosine can base pair with any natural bases with a preference toward C. Thus, A‐to‐I editing may directly change the amino acid sequence of the translated protein, which could have a global impact on the cells, depending upon the function of the protein. This editing can affect RNA splicing in eukaryotes if the A to I conversion happens at RNA splicing sites. Moreover, editing can affect the miRNA binding sites that regulate mRNA degradation and modulate mRNA abundance (Brummer et al., 2017; Nishikura, 2016). This modification is shown to have a significant contribution and relevance to neural development and neural diseases. For instance, protein‐coding sequences of glutamate receptor GRIA2 and serotonin receptor HTR2C, are edited leading to striking alterations of protein functions (Chalk et al., 2019).

### 2.2 RNA modifications on non‐coding RNAs

**FIGURE 2:**
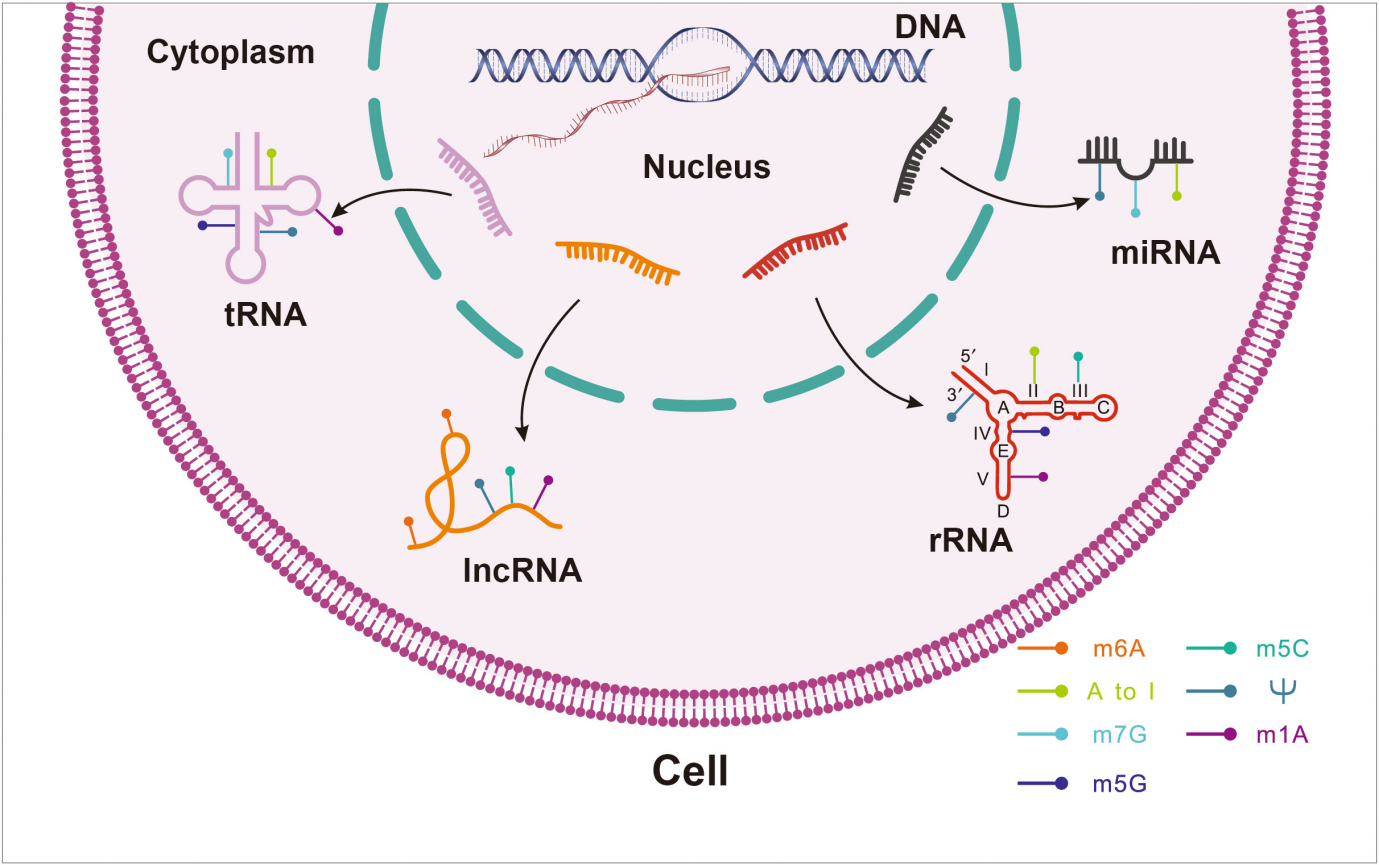
Classical RNA modification types in non‐coding RNAs

In the last decades, after the discovery of the first noninfrastructural non‐coding RNA molecules lin‐4 in 1993 (Lee et al., 1993), an explosion of studies has suggested the critical functions of non‐coding RNAs in various biological processes and human diseases. Although the regulatory modifications that control non‐coding RNA transcription at the genomic level were well established, the chemical modifications at the RNA level that control the function of non‐coding RNAs, have only recently begun to emerge (Figure 2).

#### 2.2.1 Modifications on miRNAs

miRNAs are small single‐stranded non‐coding RNAs (20 nt ~ 22 nt) that suppress gene expression post‐transcriptionally. Emerging evidence shows that they harbor multiple RNA modifications, which are catalyzed by the same enzymes as other RNA species. These modifications regulate their biogenesis, stability, and base‐pairing with targets. After transcription, the biogenesis of microRNAs starts with the processing of primary microRNAs (pri‐miRNAs) by the microprocessor complex formed by RNA‐binding protein DGCR8 and the type III RNase DROSHA. In 2015, Alarcon et al. discovered that in mammalian cells, METTL3 methylates pri‐miRNAs and marks them for recognition and processing by DGCR8, promoting miRNA maturation. Another methylation modification, “internal m7G,” catalyzed by METTL1, has been found to occur on pri‐miRNA and affects precursor‐miRNA processing. Notably, precursor‐miRNA is a double‐stranded RNA specie, making them potential substrates for adenosine deaminases acting on RNA (ADAR) enzymes. As miRNA's function is dependent on their base pairing with the target mRNA, A‐to‐I editing in miRNAs may modulate their target specificity, resulting in decreased suppressing efficiency of one or more downstream target genes. Other modifications such as m5C and pseudouridine can also affect the binding of miRNAs to targets (De Paolis et al., 2021; Han et al., 2021; Zhang et al., 2016).

#### 2.2.2 Modifications on lncRNAs

Long non‐coding RNAs or lncRNA are defined as transcripts longer than 200 nucleotides and not translated into functional proteins. Various functions and the importance of lncRNAs are getting recognized with the advancement of techniques such as next generation sequencing and exponential growth in our understanding of the genome. Depending upon the cellular localization and specific interactions with DNA, RNA, and proteins, lncRNAs can change the stability and translation of cytoplasmic mRNAs, modulate chromatin function, regulate and/or be involved in the assembly of certain complexes, interfere with signaling pathways (Statello et al., 2021). In contrast, their chemical modifications have not been well explored. MALAT‐1, XIST, and HOX are some of the relatively well‐studied examples of RNA modifications in lncRNAs. High‐throughput methods revealed that in humans both m6A and m5C were mapped to lncRNAs (Squires et al., 2012). WTAP and METTL16, components of the m6A “writer” complexes can interact with certain lncRNAs whereas methyltransferases of the m5C modification in lncRNAs are not very clear (Dinescu et al., 2019). The tRNA m5C methyltransferase NSUN2 has been identified as the writer responsible for m5C methylation in several lncRNAs (Hussain et al., 2013; Khoddami & Cairns, 2013). Another methylation modification is the m1A, despite its proof of existence in lncRNAs, the specific writers are yet to be identified. Apart from methylation, ψ also occurs in lncRNA and is also catalyzed by PUS.

#### 2.2.3 Modifications on tRNAs

Transfer RNA species have a typical and distinctive cloverleaf secondary structure (Holley et al., 1965). Unlike mRNAs, tRNAs do not encode proteins but are the direct decoder of codons in mRNAs. They are the connecting link between coding information in nucleotides and amino acids in translated proteins. They are the most heavily modified RNA molecules in terms of quantity and diversity. About 1 out of 5 nucleotides are modified in mammalian tRNAs. Recent studies have shown the importance of RNA modifications at certain positions for tRNA function in key developmental processes. Notably, in animal cells, there are two sets of tRNAs, cytoplasmic tRNAs transcribed from the nuclear genome and the mitochondrial tRNAs transcribed from the mitochondrial genome. In some cases, modifications on different sets of tRNAs are carried out by different enzymes. On the contrary, the same type of modifications on different tRNA species will have different downstream targets and biological effects. A huge variety of RNA modifications have been found on tRNAs, such as N2‐methylguanosine (m2G), N2,N2‐dimethylguanosine (m22G), 1‐methyl‐guanosine (m1G), N4‐acetylcytidine (ac4C), m1A, dihydrouridine, 3‐(3‐amino‐3‐carboxypropyl)uridine (acp3U), 3‐methylcytidine (m3C), inosine, 1‐methyl‐inosine, 5‐methoxycarbonylmethyluridine (mcm5U), 5‐methoxycarbonylmethyl‐2‐thiouridine (mcm5s2U), queuosine, galactosyl‐queuosine (gal Q), mannosyl‐queuosine (manQ), 5‐formyl‐20 ‐O‐methylcytidine (f5Cm), N6‐threonylcarbamoyladenosine (t6A), 2‐methylthio‐ N6‐threonylcarbamoyladenosine (ms2t6A), N6‐methyl‐N6‐threonylcarbamoyladenosine (m6t6A), N6‐ isopentenyladenosine(i6A), peroxywybutosine (o2yW), wybutosine (yW), 1‐methylpseudouridine (m1J), 20‐O‐methylpseudouridine (Jm), m7G, m5C, 2‐methyladenosine (2 mA), and 5‐methyluridine (5 mU) (de Crecy‐Lagard et al., 2019; Juhling et al., 2009). All these modifications can be grouped based on their location, into 2 major types—modifications in the anti‐codon loop, especially at the wobble position, and modifications outside the anticodon loop. For modifications within the anti‐codon loop, RNA modifications can directly affect the decoding of the codons into amino acids by affecting the base pairing of the codon in mRNAs and tRNA‐carrying amino acids (Suzuki, 2021). For modifications outside of the loop, they are most likely to influence the secondary structure of tRNAs.

#### 2.2.4 Modifications on rRNAs

Like tRNAs, rRNAs are non‐coding but are directly involved in the process of translation. rRNA modifications are dense but lack diversity. Only about 2% of the nucleotides in rRNAs are modified, with most of them being 2’‐OMe on the ribose sugar and isomerization of uridine. Modifications on the base, such as methylation, also exist. As the 2′ hydrophilic hydroxyl group on the ribose is what discriminates RNA bases from DNA bases, the 2’‐OMe modification of RNA can have fundamentally altering effects on the structure and stability of RNA and even in the biogenesis of ribosomes. Yildirim et al. showed that 2’‐OMe enhances duplex stability of RNA–RNA hybrids (Yildirim et al., 2014).

Despite the conservation of rRNA and ribosomal structure, composition, and function in both prokaryotes and eukaryotes, there are dichotomies in rRNA modifying machinery. In E. coli, modifications are carried out by pure protein enzymes that are site or region‐specific whereas 2’‐O‐methylation and pseudouridylation in eukaryotes are done via site‐specific small nucleolar RNA–protein complexes. Site specificity is determined by the small nucleolar RNAs (snoRNAs) through base pairing and it guides the modifying enzymes to the modification sites; Box C/D snoRNAs guide 2’‐OMe and Box H/ACA snoRNAs guide pseudouridylation (Watkins & Bohnsack, 2012). RNA modifications in rRNA are not randomly distributed. Instead, they are mostly hidden inside the ribosome and occur in conserved sites and functional sites, such as the A, P, and E sites for tRNA binding, mRNA binding, and the peptidyl transfer center, respectively. The former feature suggests that most modifications happen before the ribosome assembly and once assembled, they are not easily accessible to the demodifying or remodifying enzymes if any. It is well appreciated that demodifying enzymes unlikely exist and change rRNA modifications in mature ribosomes. The latter feature implies that these modifications may have a global impact within the cell (Decatur & Fournier, 2002; Roundtree et al., 2017). Nonetheless, inducible modifications do exist. In yeast, it has been observed that post‐diauxic growth and heat‐shock induce pseudouridylation in two rRNAs which supports the idea that these modifications are dynamic and could serve to alter ribosome function (Carlile et al., 2014; Schwartz et al., 2014).

## 3 RNA MODIFICATIONS IN AGING‐RELATED NEURODEGENERATIVE DISEASES

Aging‐related neurodegenerative diseases include Alzheimer's disease (AD), Parkinson's disease (PD), amyotrophic lateral sclerosis (ALS), stroke, and frontotemporal degeneration. Accumulating evidence has indicated that age‐related neurodegenerative diseases result from various reasons. Among them, epigenetic changes especially RNA modification that could have serious implications in this aspect should be further explored.

### 3.1 AD and RNA modification

AD was first described by Alois Alzheimer in 1906. It is one of the most damaging aging‐related neurodegenerative diseases, the prevalence of which increases as the global population ages (Cummings et al., 2014). Despite its clinical importance, effective therapy against AD is yet to be identified. The core clinical manifestation of AD is the loss of synaptic plasticity, which is closely related to the decline in cognitive ability. Some of the initial clinical features of AD are defects in the capability of creating and storing new memories (Soria Lopez et al., 2019). Although AD was first described almost a century ago, the pathogenesis of AD though understood better than before, requires further investigation.

m6A is an abundant RNA modification in the brain, and recent studies have demonstrated that the m6A methylation of RNA could promote the development of AD. By using m6A‐sequencing together with high‐throughput liquid chromatography–tandem mass spectrometry (LC–MS/MS), it is found that the expression level of METTL3 was significantly reduced along with m6A levels in 5xFAD mice when compared with control mice (Shafik et al., 2021). Consistently, the significantly decreased neuronal m6A levels and METTL3 expression were also observed by immunoblot analysis in human AD brains compared with the age‐matched control cases (Zhao et al., 2021). Knockdown of METTL3 in the mouse hippocampus caused memory loss, neurodegeneration, spine loss, and gliosis (Zhao et al., 2021). Mechanistically, METTL3 deficiency delays the mRNA degradation of m6A‐modified cell cycle genes, including Cyclin D1 and Cyclin D2, in the hippocampus and in primary neuron cultures, which causes dysregulated cell cycle and oxidative stress (Zhao et al., 2021). In addition, a recent study examining the expression profiles of m6A‐regulated genes in human AD post‐mortem brains has reported the aberrant expression of METTL3 and RBM15B in the AD hippocampus and indicated that the accumulation of METTL3 in the insoluble fractions positively correlated with that of Tau in hippocampal lysates, suggesting that potential perturbations in m6A signaling may contribute towards neuronal dysfunction in AD (Huang et al., 2020). Diabetes and obesity are thought to be closely related to AD. It is found that FTO activates mTOR signaling and reduces the mRNA level of TSC1, therefore activating the phosphorylation of Tau in insulin defects‐associated AD, and conditional knockout of FTO in the neurons reduces the cognitive deficits in 3xTg AD mice (Li et al., 2018). Besides, the NIA‐LOAD study identified a genetic variant in the FTO gene loci significantly associated with AD, and FTO expression was significantly lower in the cortex and amygdala tissues of AD patients compared with controls, suggesting the functional role of FTO in Alzheimer's Disease (Reitz et al., 2012). This is further confirmed by a prospective study showing FTO AA‐genotype posed a higher risk for AD and dementia (Keller et al., 2011). These findings suggest that m6A modification may play a pivotal role in the pathogenesis and progression of AD.

LC–MS/MS revealed that small RNA modifications of AD patients changed compared with normal controls, including 2’‐O‐methylcytidine (Cm), m7G, and 2’‐O‐methylguanosine, were increased while N2, N2,7‐trimethylguanosine (m2,2,7G), and N2,N2‐dimethylguanosine were dramatically decreased in the 15‐25 nt RNA fraction from the cortex of AD brains. RNA‐seq analysis revealed that most of these fractions are miRNAs. Interestingly, in the 30–40‐nt small RNA fraction, Cm, 2’‐O‐methyluridine (Um), and m7G modifications showed higher levels compared with controls whereas m1G, m2,2,7G, and pseudouridine modifications were reduced (Figure 1). Among them, tRNA‐derived small RNAs, rRNA‐derived small RNAs, Y RNA‐derived small RNAs, and other unannotated RNAs were part of the major fractions in RNA‐seq analysis (Zhang, Trebak, et al., 2020). By using microfluidic‐based high‐throughput PCR along with next‐generation sequencing, it was found that A‐to‐I RNA editing levels were reduced in Alzheimer's disease samples when compared with controls (Khermesh et al., 2016). Similarly, the A‐to‐I RNA editing events of AD were systematically annotated (Wu et al., 2021). 1,676,363 editing sites were detected in 1524 samples across 9 brain regions from ROSMAP, MayoRNAseq, and MSBB studies, within which 108,010 and 26,168 editing events were identified to promote or inhibit AD progression, respectively (Wu et al., 2021). 5582 brain region‐specific editing events with potentially dual roles in AD across different brain regions were also noticed (Wu et al., 2021).

### 3.2 ALS and RNA modification

ALS is one of the most common age‐related neurodegenerative diseases characterized by progressive weakness and muscle atrophy, causing damage to upper and lower motor neurons (Oskarsson et al., 2018). The pathogenesis of amyotrophic lateral sclerosis involves several mechanisms, among which RNA modifications deserve a more thorough investigation.

ADAR2 specifically catalyzes A‐to‐I RNA editing at the glutamine/arginine (Q/R) site of GluA2, a subunit in the majority of AMPA receptors in the adult brain, and changes the glutamine (position 607; encoded by CAG) to an arginine (edited to CIG and translated as CGG) within the ion pore of GluA2, which is indispensable for normal AMPA receptor function (Sommer et al., 1991). In conditional ADAR2 knockout mice, the Q/R site of GluA2 cannot be edited by ADAR2, which results in the slow death of the motor neurons (Hideyama et al., 2010; Hideyama & Kwak, 2011). Recent evidence demonstrated that the efficiency of RNA editing at the GluA2 Q/R site was significantly lower in all ALS cases compared with that of the control subjects. Interestingly, of the three members of the ADAR family, only the enzymatic activity of ADAR2 was downregulated in ALS motor neurons, which suggests that once ADAR2 expression levels decreased below the required threshold to edit all GluA2 Q/R sites, motor neurons enter a death cascade. It is also suggested that the progressive downregulation of ADAR2 may be closely related to the pathogenesis of ALS, wherein the failure of A‐to‐I transition at the GluA2 Q/R locus is critical (Hideyama et al., 2012). Furthermore, the CYFIP2 mRNA K/E site was predominantly edited by ADAR2 and has been newly identified as ADAR‐mediated A‐to‐I editing positions, which could provide a clue to the pathogenesis of ALS (Kwak et al., 2008). Notably, a deeper understanding of the various RNA modifications may shine light on the mechanism‐based therapeutic approach to treating age‐related neurodegenerative diseases. Similarly, glutamate receptor GRIA2 editing is significantly reduced in the motor neurons of ALS patients as well as in patients with schizophrenia and bipolar disorder. Reduced editing is accompanied by reduced ADAR2 expression in these patients, with RNA editing deficiency contributing to motor neuron toxicity in ALS (Maas et al., 2006). Moreover, TDP‐43, a pathological hallmark of ALS, is exclusively expressed in motor neurons lacking ADAR2 in patients with sporadic ALS (Aizawa et al., 2010), demonstrating the pathogenic role of unedited GluA2 at the Q/R locus in ALS.

## 4 RNA MODIFICATIONS IN AGING‐RELATED CARDIOVASCULAR DISEASES (CVDS)

CVDs remain the leading cause of death globally (Mortality, & Causes of Death, C, 2013), and biological aging is a major risk factor in CVDs, such as atherosclerosis, coronary heart disease, myocardial infarction, hypertension, stroke, cardiac hypertrophy, and heart failure (HF) (North & Sinclair, 2012). By 2030, approximately 20% of the population will be 65 years of age or older. By that time, CVDs will be responsible for 40% of deaths and the cost of treating CVDs will have tripled (Fleg et al., 2011; Heidenreich et al., 2011). Therefore, it is critical to understand the underlying mechanism by which aging acts as an important determinant of the etiology of CVDs. Here, we discuss some of the associations between RNA modifications and age‐related CVDs.

### 4.1 Atherosclerosis

Atherosclerosis is a chronic inflammatory disease that progresses slowly with the accumulation of cholesterol, lipids, and cellular debris in blood vessels, which in turn greatly increases the risk of restricting blood flow and rupture of blood vessels, contributing to the development of heart attack (myocardial infarction) and stroke (Gistera & Hansson, 2017). Aging‐related obesity is the predominant risk factor for atherosclerosis. New evidence revealed that zinc‐finger protein 217 (ZFP217) can reduce the expression of m6A by activating the expression of FTO, which ultimately leads to increased adipogenesis and obesity (Song et al., 2019). Recent studies have also demonstrated that METTL3 plays a crucial role in atherogenesis induced by oxidative stress and disturbed blood flow. METTL3‐mediated RNA hypermethylation stabilized NLRP1 mRNA and degraded KLF4 mRNA through YTHDF1 and YTHDF2 m6A reader proteins, respectively (Chien et al., 2021). Knockdown of METTL3 restored the mRNA levels of NLRP1 and KLF4 and prevented the atherogenic process (Chien et al., 2021). In addition, METTL14 was demonstrated to directly bind to FOXO1 mRNA and enhance FOXO1 mRNA translation by increasing its m6A modification, which thereby increases adhesion molecule expression, aggravating endothelial inflammation, and contributes to atherosclerosis development (Jian et al., 2020). Silencing METTL14 also inhibited the proliferation and invasion of atherosclerotic vascular endothelial cells (ASVECs) by inhibiting the expression of miR‐19a which promotes the proliferation and invasion of ASVECs (Zhang et al., 2020).

ADAR1 is known to bind to a vast majority of the double‐stranded RNAs and catalyzes the deamination of adenosine to inosine (A‐to‐I) (Stellos et al., 2016). Notably, ADAR1, the main RNA editor in endothelial cells, could enhance the stability of Cathepsin S (CTSS) and its expression by inducing A‐to‐I RNA editing in Alu elements in the 3'UTR of CTSS transcripts and recruiting HuR to the 3’UTR (Stellos et al., 2016). Moreover, analysis of microarray‐based data and results of immunohistochemistry revealed that the expression levels of both CTSS and ADAR1 were upregulated in atherosclerotic carotid plaques. Besides, ADAR1‐induced A‐to‐I RNA editing could stabilize the atherosclerosis‐associated NEAT1 lncRNA expression (Vlachogiannis et al., 2021).

### 4.2 Cardiac hypertrophy

While hypertrophy initially develops as an adaptive response to physiological and pathological stimuli, both physiological and pathological hypertrophy involves the enlargement of individual cardiomyocytes. Nearly a quarter of the transcripts in both mouse and human hearts showed m6A RNA methylation, and an increasing number of studies provide clear evidence that RNA modifications play important roles in cardiac hypertrophy (Kumari et al., 2021). Thus, a better understanding of the underlying molecular mechanisms that regulate cardiac hypertrophy would give better insight into novel therapeutic approaches.

With m6A sequencing, several studies have demonstrated that m6A methylation levels are markedly increased in cardiac hypertrophy. METTL3 could catalyze m6A methylation on specific mRNA subpopulations that drive cardiomyocyte hypertrophy. Both knockdown and overexpression of METTL3 in vitro and in vivo affect cell size and cell remodeling and the inhibition of METTL3 has been shown to be sufficient to prevent hypertrophy *in vitro* (Dorn et al., 2019 *;* Kmietczyk et al., 2019 *)*. These findings underscore the significance of this novel mechanism of cardiac hypertrophy. It is well known that leptin directly induces cardiomyocyte hypertrophy. Recently, Gan et al. demonstrated that the expression levels of FTO were upregulated after leptin treatment in cultured myocytes, suggesting its potential role in mediating the hypertrophic response to leptin (Gan et al., 2013). Similarly, FTO knockdown can blunt the hypertrophy of neonatal rat cardiomyocytes induced by α‐adrenergic stimulation with phenylephrine (Kmietczyk et al., 2019). These findings suggest that the effects of FTO on cardiac hypertrophy may be stimulation‐dependent, in addition, the detailed molecular mechanism needs to be further clarified. Moreover, whether the demethylase activity of FTO is necessary for this function or not, requires further investigation.

Meanwhile, non‐coding RNAs specifically expressed in the heart could participate in molecular networks associated with myocardial hypertrophy, including piRNAs. A recent study found that the expression level of DQ726659, an uncharacterized piRNA named cardiac‐hypertrophy‐associated piRNA (CHAPIR), was significantly increased in TAC‐induced hypertrophic mouse hearts. CHAPIR directly interacted with METTL3 which suppresses the m6A modification of Parp10 mRNAs thereby increasing Parp10 protein levels and promoting NFATC4‐induced pathological cardiac hypertrophy (Gao et al., 2020). Similarly, cardiac‐specific targets of miR‐133a were enriched in m6A modifications. IGF2BP2, a key m6A reader, was observed to promote the assembly of the m6A‐modified miR‐133a‐AGO2‐RISC complex on the mRNA targets of miR‐133a, thereby enhancing the inhibitory effect of miR‐133a and protecting from cardiac hypertrophy (Qian et al., 2021).

### 4.3 RNA modification and other CVDs

Hypertension, one of the most important risk factors for CVDs, is usually defined as a prolonged increase in systemic arterial pressure above a certain threshold (Giles et al., 2009). The incidence of hypertension rises dramatically with age and 70% of older adults have hypertension in 2015 (Mozaffarian et al., 2015). Interestingly, 33 (2.67%) m6A‐associated single‐nucleotide polymorphisms (m6A‐SNPs) were found to be significantly associated with blood pressure in three genome‐wide association studies from East Asian populations, within which rs56001051 (C1orf167) and rs197922 (GOSR2) were significantly associated with hypertension (Mo et al., 2019a). Another study genotyped 217 individuals (86 men and 131 women) with hypertension and found FTO rs9939609 had a negative association with blood pressure in male hypertensive patients (Marcadenti et al., 2013).

The prevalence of HF, characterized as reduced cardiac function and left ventricular dilatation, is predicted to increase remarkably by 46% in the United States from 2012 to 2030 due to the aging population (Heidenreich et al., 2013). Recently, emerging studies have revealed the regulatory mechanisms of RNA methylation in the pathogenesis of HF (Komal et al., 2021). m6A‐sequencing and transcriptome analysis of the heart tissues from human HF patients and mouse transverse aortic constriction (TAC) models revealed that m6A modification profiles were changed more dramatically than gene expression and RNAs with altered m6A modification were mainly enriched in metabolic and regulatory pathways (Berulava et al., 2020). Furthermore, cardiomyocyte‐specific deletion of FTO accelerated the progression of HF after TAC surgery (Berulava et al., 2020). Cardiomyocyte‐specific deletion of ADAR1 leads to an excessive amount of cardiomyocyte loss, resulting in cardiac dysfunction and eventual lethality. Lack of ADAR1 leads to a global reduction in miRNA production, in particular of miR‐199a‐5p, and the activation of unfolded protein response (UPR)‐driven apoptotic response, which hampers ER stress handling in cardiomyocytes. Inhibition of the UPR in ADAR1‐knockout hearts significantly reduced cardiomyocyte loss and restored survival of the animals due to improved cardiac function, which pointed to an essential role for ADAR1 in cardiomyocyte survival and maintenance of cardiac function (El Azzouzi et al., 2020). Interestingly, it is also found that HF patients with reduced ejection fraction had higher concentrations of pseudouridine in plasma compared with healthy controls (Alexander et al., 2011). These studies suggest that RNA modification may serve as therapeutical target against HF.

Stroke is the second most common cause of CVD‐related mortality and the number of strokes and deaths due to stroke increases substantially each year (Collaborators, 2021). Stroke was found to significantly increase the global m6A methylation levels in mouse cortex after reperfusion of transient focal ischemia and the genome‐wide analysis of the methylated RNAs showed that 147 transcripts (127 mRNAs and 20 lncRNAs) have altered m6A levels, among which 95% (122 mRNAs and 17 lncRNAs) were significantly hypermethylated after stroke, which may due to the downregulation of FTO (Chokkalla et al., 2019). The integrative analysis of the association between m6A‐SNPs and ischemic stroke found that 310 (7.39%) m6A‐SNPs were nominally associated with ischemic stroke (Mo et al., 2019b). In addition, another study revealed that YTHDC1 expression was upregulated in the early phase of ischemic stroke, and overexpression of YTHDC1 significantly decreased brain infarct volume (Zhang, Wang, et al., 2020). Mechanistically, YTHDC1 activated Akt phosphorylation via promoting the degradation of m6A‐modified PTEN mRNA (Zhang, Wang, et al., 2020).

Taken together, these studies suggested that epigenetic modifications of mRNAs have a great impact on aging‐related CVDs which could provide critical clues to developing future therapies against age‐related CVDs.

## 5 RNA MODIFICATIONS IN AGING‐RELATED CATARACTS

Aging‐related cataracts are the main disease of visual impairment and blindness in the world, which commonly occur in people over 50 years old. Cataracts are formed due to the decrease in transparency of the lens (Yang et al., 2019), and so far, the only available treatment for cataracts is surgery (Dubois & Bastawrous, 2017). Therefore, research into the mechanism of cataracts is urgently required to find potential targets for novel therapeutics. A recent study investigated the involvement of m6A circRNAs and methyltransferases in the lens epithelium cells(LECs). By performing genome‐wide profiling of m6A‐modified circRNAs in lens epithelium cells, they found 2472 m6A peak distributions on 1248 circRNAs with the up‐methylation degree and 2174 m6A peaks distribution on 1148 circRNAs with the down‐methylation degree. Moreover, the expression of m6A‐modified circRNAs in the age‐related cataract LECs was lower than that of the controls, which strengthened the dynamic relationship between the m6A modifications at the circRNAs and expression of m6A circRNAs in age‐related cataract LECs. They also examined the expression levels of a key methyltransferase, ALKBH5, and two major methyltransferases, METTL3 and WTAP. It was found that the mRNA expression levels of ALKBH5 were significantly upregulated when compared with the control groups, suggesting that ALKBH5 decreases the m6A modifications of circRNAs (Li, Yu, et al., 2020). Interestingly, METTL3 could modulate the proliferation and apoptosis of LECs in diabetic cataracts by targeting the 3’UTR of ICAM‐1 and stabilizing its mRNA stability (Yang et al., 2020).

Isomerization of uridine to pseudouridine is one of the most abundant RNA modifications and is catalyzed by the H/ACA small ribonucleoprotein complex that is composed of four core proteins, dyskerin (DKC1), NOP10, NHP2, and GAR1. Histological analysis has revealed that in Dkc1^elu1/elu1^ mutant larvae show microphthalmia and cataracts with abnormal eyes and retinas, accompanied by a large number of cells with neuroepithelial properties (Balogh et al., 2020). Using MeRIP‐seq and RNA‐seq, one recent study comprehensively analyzed the transcriptome‐wide m6A methylome and gene expressions of the anterior capsule of the lens in highly myopic patients with nuclear cataract anterior. They found that METTL14 was upregulated whereas METTL3, FTO, ALKBH5, YTHDF1, and YTHDF2 were downregulated, which suggests that m6A methylation was strongly associated with the pathogenic mechanism of high myopia (Wen et al., 2021). Overall, these studies have uncovered the regulatory roles of m6A modifications in aging‐related cataracts.

## 6 RNA MODIFICATIONS IN AGING‐RELATED OSTEOPOROSIS

Aging‐related osteoporosis is characterized by low bone mass and over‐accumulation of fatty tissue in the bone marrow environment that increases the risk of fracture (Duque et al., 2009). With aging, the composition of the bone marrow shifts to favor the presence of adipocytes, osteoclast activity increases and osteoblast function declines, leading to osteoporosis (Coughlan & Dockery, 2014). METTL3, the key methyltransferase of m6A, was observed to regulate the fate of osteoporosis. Firstly, the expression levels of METTL3 and m6A methylation are significantly decreased in both osteoporosis patients and mouse models (Yan et al., 2020). Downregulation of METTL3 caused the decline in bone formation and overexpressed METTL3 could partially rescue the feature of osteoporosis such as reduction in bone formation. Molecularly, METTL3 mediates m6A methylation of RUNX2, a key factor involved in osteogenesis, and enhances its cellular stability (Yan et al., 2020). METTL3 knockout reduced the translation efficiency of MSCs lineage allocator Pth1r and then led to a reduction of the global methylation level of m6A and disruption of the PTH‐induced osteogenic and adipogenic responses, which eventually affects the osteogenic and adipogenic differentiation of mesenchymal stem cells (Wu et al., 2018). Apart from this, a recent study revealed different molecular mechanisms of METTL3‐dependent m6A modification in osteoclast differentiation. Here, the depletion of either METTL3 or YTHDF2 promoted the stability and the expression of Atp6v0d2 mRNA (Li, Cai, et al., 2020). Besides, knockdown of METTL3 reduces the expression level of VEGFA and its splice variants, VEGFA‐164 and VEGFA‐188 thereby regulating osteogenic differentiation (Tian et al., 2019). More interestingly, METTL3 can also regulate osteogenic differentiation by promoting m6A methylation modifications of the critical upstream regulator of NF‐κB signaling: MYD88‐RNA, which in turn triggers the activation of NF‐κB, thereby inhibiting osteogenic progression (Yu et al., 2020). m6A methylation is also catalyzed by METTL14 which serves as the RNAbinding scaffold that recognizes the substrate. Recent studies demonstrated the critical roles of the miR‐103‐3p/METTL14/m6A signaling axis in osteoblast activity. Here, miR‐103‐3p inhibits osteoblast activity by directly targeting METTL14 while METTL14‐dependent m6A methylation enhances the recognition of pri‐miR‐103‐3p by DGCR8 and the subsequent processing into mature miR‐103‐3p, thereby modulating osteoblast activity (Sun, Wang, et al., 2021).

FTO is a key regulator associated with adipogenesis, and the complete depletion of FTO in mice results in postnatal growth retardation. FTO knockout mice have not only a significantly shorter body length over the lifetime but also a much lower bone mineral density (Gao et al., 2010). To evaluate the effect of FTO on bone mass and to prevent the potential confounding effect of FTO on global metabolism and body composition, mice lacking FTO selectively in osteoblasts (FTO^Oc KO^) were generated. These mice showed a significant decrease in bone volume and trabecular number at 30 weeks of age. Furthermore, the results of static and dynamic histomorphometric analyses showed that the bone formation rate in mutant mice was decreased by 66% with bone marrow adipocyte number per bone marrow area being increased when compared with controls. FTO functioned through demethylating and then enhancing the stability of the mRNAs of Hspa1a and other genes that can protect cells from genotoxic damage (Zhang et al., 2019). The above results implied that FTO is required for the maintenance of bone mass and FTO^Oc KO^ mice manifest the phenotype consistent with age‐related bone loss. miR‐149‐3p was found to directly target FTO mRNA and modulate the adipogenic differentiation of bone marrow‐derived mesenchymal stem cells (Li et al., 2019). Besides, during aging and osteoporosis, FTO was upregulated by GDF11 in both humans and mice, and then stabilized the Pparg mRNA through the demethylation of m6A, leading to the differentiation of bone mesenchymal stem cells to adipocytes rather than osteoblasts (Shen et al., 2018). FTO also plays an intrinsic role in osteoblasts by enhancing the stability of mRNAs of proteins that can protect cells from genotoxic damage via Hspa1a‐NF‐κB signaling (Zhang et al., 2019). Altogether, these findings provide new perspectives on the pivotal role of m6A in regulating age‐related bone diseases such as osteoporosis.

## 7 RNA MODIFICATIONS IN AGING‐RELATED FERTILITY DECLINE

Age‐related fertility decline is inevitable and irreversible, especially for female reproductive potential. Several demographic and epidemiological studies have long recognized that female fertility declines with age, most notably the decline in ovarian function (Leridon, 2004; Menken et al., 1986; Nelson et al., 2013). The mechanisms involved in the process of ovarian aging have gained increased attention and focus. Herein, we highlight the importance of RNA modification in ovarian aging.

All m6A modifications of the granulosa cells of aged human ovaries were measured in order to investigate the relationship between ovarian aging and m6A modification. It was found the level of m6A modifications was significantly increased and the expression level of FTO was downregulated. m6A sequencing showed that increased m6A in the 3′UTR of FOS mRNAs resulted in reinforcing the stability of FOS mRNAs (Jiang et al., 2021). Another study also concurrently found that the expression of FTO decreased and the content of m6A increased with aging in human follicular fluid, granulosa cells, and mouse ovary (Sun, Zhang, et al., 2021). Besides, the chemotherapy drug, cyclophosphamide, could increase the m6A level and significantly inhibit the expression levels of RNA demethylase FTO in a time‐ and concentration‐dependent manner, which is further associated with premature ovarian aging (Huang et al., 2019). More research exploring the precise mechanism of RNA modifications in ovarian aging would give insight into possible strategies to postpone ovarian aging.

## 8 CONCLUSION AND PERSPECTIVES

**FIGURE 3:**
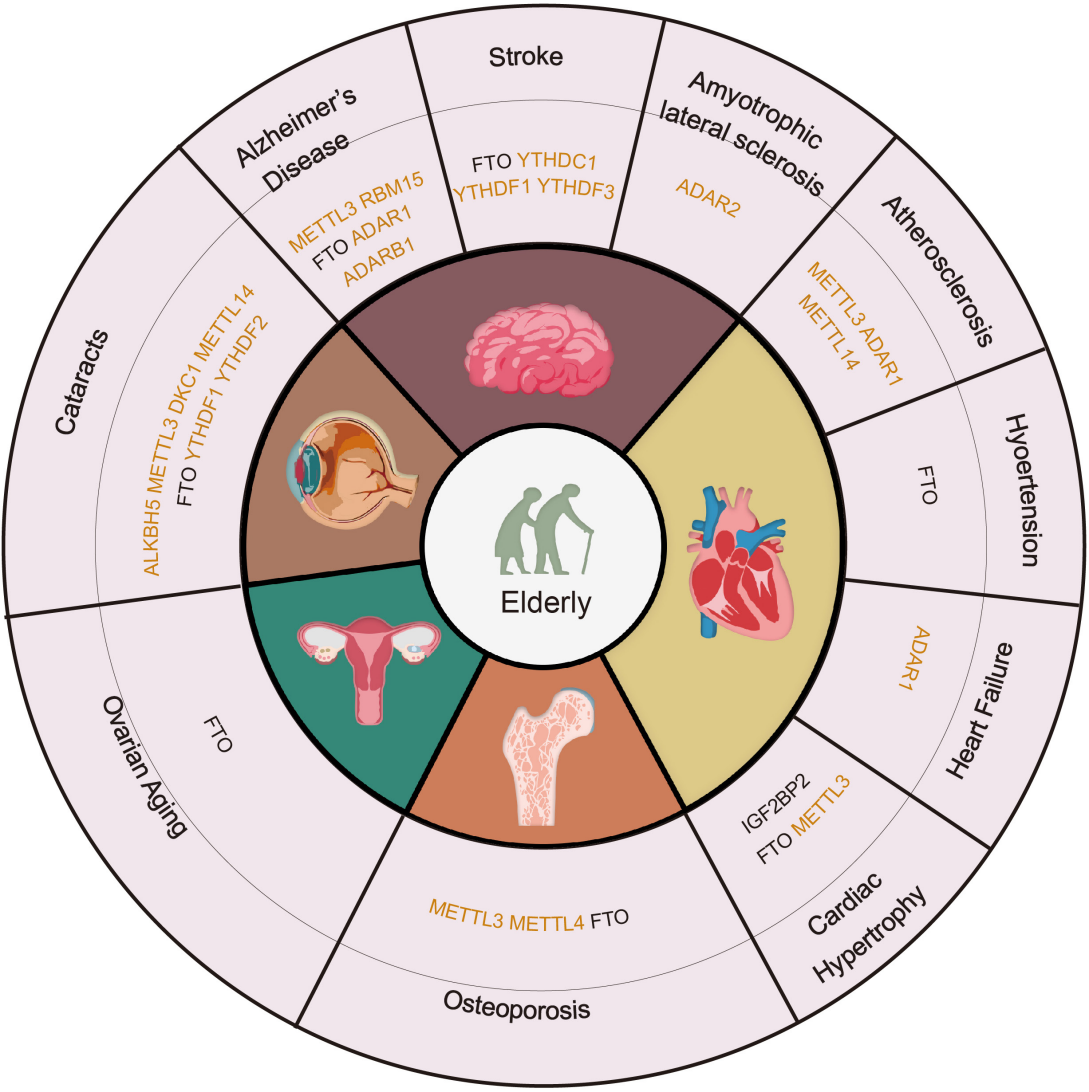
RNA modification genes associated with aging‐related diseases

In conclusion, the field of epitranscriptomics has emerged rapidly in recent years and RNA modifications have been emerging as a new focus and novel therapeutical targets against aging‐related diseases. In this review, we have summarized RNA epitranscriptomic regulation and the mechanisms involved in aging‐related diseases. We found that RNA modification is involved in many diseases that are aging‐related, and plays an essential role in impacting mRNA stability, translation, and control of protein levels of key genes that are involved in pertinent disease‐associated pathways (Table 1). Collectively, we have summarized several studies that highlight the crucial role of RNA modification in aging‐related diseases (Figure 3). Understanding their functions in the context of aging‐related disease could provide newer perspectives that enrich the theoretical basis of aging‐related disease. However, a few more points need to be further thoroughly analyzed. (1) The epigenetic mechanisms of aging‐related diseases have predominantly focused on DNA methylation, histone modifications, and chromatin rearrangement, while the important biological functions of m6A modification have been ignored and require thorough future exploration. (2) Although m6A modification is increasingly being studied in other fields, there are still many modifying erasers/writers/readers that remain to be discovered. Their functions and potential therapeutic implications in aging‐associated diseases have yet to be investigated. Moreover, few studies that have focused on m6A application and m6A‐targeting drug therapy need to be further explored in‐depth. (3) PD is another common progressive neurodegenerative disorder, which is characterized by rigidity, bradykinesia, tremor, and gait disturbances (Jankovic, 2008). In addition to DNA methylation and chromatin remodeling, a recent work identified m6A‐modifying genes (including METTL3, METTL14, WTAP, FTO, ALKBH5, YTHDF1, YTHDF2, YTHDF3, HNRNPC, and ELAVL1) in a total of 1647 sporadic PD patients, among them were 214 rare variants in these 10 m6A‐modification genes and 16 common variants in seven genes. Although an apparent association of 10 m6A‐modification genes and sporadic PD in the Chinese cohort was not found, further functional studies are needed to explore the association between RNA modifications and PD, given the impact of RNA modification on brain development and other aging‐related neurological disorders (Qin et al., 2020). There are very few studies on RNA modification related to macular degeneration and the regulation of gut microbes, which are areas worth exploring to gain more knowledge and advance the field of aging‐associated RNA modifications. Taken together, further efforts are required to gain an in‐depth insight into the role of RNA modifications in aging‐related diseases and would provide new potential molecular targets for research and development of pharmacological and clinical therapies for many aging‐related diseases.

**TABLE 1:** The regulation and roles of RNA modifications in aging‐related diseases

Category of diseases	Pathogenic phenotype	RNA modification	Gene expression alterations	Molecular consequences of altered RNA modification/pathogenic relevance	References
Alzheimer's disease (AD)	AD	m6A	Decreased METTL3 expression	Many AD‐related transcripts exhibit decreased m6A modification, which is correlated with reduced protein levels.	(Shafik et al., 2021)
	AD	m6A	Decreased METTL3 expression	METTL3 depletion resulted in elevated levels of m6A‐modified CCND2	(Zhao et al., 2021)
	AD	m6A	Decreased METTL3 expression and increased RBM15B expression	Significant correlation with the expression level of insoluble Tau protein in the postmortem of human AD	(Huang et al., 2020)
	Diabetes and obesity‐associated AD	m6A	Increased FTO expression	Promoting the activation of mTOR by increasing the mRNA level of TSC1	(Li et al., 2018)
	AD	Multiple RNA modifications	Altered modification on small RNAs, including tsRNA, rsRNAs, ysRNAs, and other unannotated RNAs	Not specified	(Zhang, Trebak, et al., 2020)
	AD	A‐to‐I editing	Aberrant expression of ADAR1 and ADARB1	Editing levels of 35 target sites within 22 genes were significantly altered in AD patients' brain tissues	(Khermesh et al., 2016)
Amyotrophic lateral sclerosis (ALS)	ALS	A‐to‐I editing	Decreased ADAR2 expression	Failure of A‐to‐I transition at GluA2 Q/R locus; reduced glutamate receptor GRIA2 editing in the motor neurons	(Hideyama et al., 2012; Kwak et al., 2008; Maas et al., 2006)
Atherosclerosis	Atherogenesis	m6A	Increased METTL3 expression	Stabilization of NLRP1 mRNA and degradation of KLF4 mRNA	(Chien et al., 2021)
	Atherosclerosis development	m6A	Increased METTL14 expression	Promoting FOXO1 translation	(Jian et al., 2020)
Atherosclerosis	The proliferation and invasion of ASVEC	m6A	Increased METTL14 expression	Promoting the maturation of miR‐19a	(Zhang et al., 2020)
	Atherosclerotic carotid plaques	A‐to‐I editing	Increased ADAR1 expression	Enhancing the stability of Cathepsin S (CTSS)	(Stellos et al., 2016)
	Atherosclerosis	A‐to‐I editing	Not specified	Stabilizing the atherosclerosis‐associated NEAT1 lncRNA expression	(Vlachogiannis et al., 2021)
Cardiac Hypertrophy	Cardiac hypertrophy	m6A	Increased METTL3 expression	Stabilizing a subpopulation of mRNAs driving cardiac hypertrophy	(Dorn et al., 2019; Kmietczyk et al., 2019)
		m6A	Increased FTO expression	Hypertrophic response to leptin	(Gan et al., 2013)
		m6A	Blockage of METTL3 function by cardiac‐hypertrophy‐associated piRNA (CHAPIR)	CHAPIR–PIWIL4 complexes block METTL3 from catalyzing m6A modification on Parp10 mRNAs, which upregulates PARP10 expression and promotes NFATC4‐dependent pathological hypertrophy	(Gao et al., 2020)
		m6A	Decreased miR‐133a expression	IGF2BP2 promotes the localization of m6A‐modified miR‐133a in AGO2‐RISC complex and enhances the function of miR‐133a	(Qian et al., 2021)
Heart Failure (HF)	Cardiac dysfunction	A‐to‐I editing	Decreased ADAR1 expression	Resulting a global reduction of miRNAs, especially miR‐199a‐5p, which activates UPR in cardiomyocytes	(El Azzouzi et al., 2020)
	HF	m6A	Not specified	Regulating RNA translation efficiency	(Berulava et al., 2020)
Stroke	Stroke	m6A	Decreased FTO expression and increased expression of YTHDF1 and YTHDF3	Altering the m6A level of 147 transcripts that are involved in inflammation, apoptosis, and transcriptional regulation	(Chokkalla et al., 2019)
	Brain infarct volume	m6A	Increased YTHDC1 expression	Facilitating the degradation of PTEN mRNA	(Zhang, Wang, et al., 2020)
Cataract	Cataract	m6A	Increased ALKBH5 expression	Decreasing the m6A modifications of circRNAs	(Li, Yu, et al., 2020)
	Proliferation and apoptosis of LECs	m6A	Increased METTL3 expression	Stabilizing the ICAM‐1 mRNA	(Yang et al., 2020)
Cataract	High myopia	m6A	Increased METTL14 expression and decreased expression of ALKBH5, METTL3, FTO, YTHDF1, and YTHDF2	Differentially methylated genes were enriched in the pathways regulating the formation of extracellular matrix.	(Wen et al., 2021)
	Cataract	pseudouridine	Decreased DKC1 expression	Defective pseudouridination of small nucleolar ribonucleoproteins	(Balogh et al., 2020)
Osteoporosis	Bone formation	m6A	Decreased METTL3 expression	Enhancing the cellular stability of RUNX2	(Yan et al., 2020)
	Osteogenic and adipogenic differentiation of MSCs	m6A	Decreased METTL3 expression	Reduced translation efficiency of Pth1r due to decreased METTL3 expression	(Wu et al., 2018)
	Osteoclast differentiation	m6A	Increased METTL3 expression	METTL3 deficiency promotes the stability and the expression of Atp6v0d2 mRNA and reduced the expression level of Vegfa and its splice variants	(Li, Cai, et al., 2020; Tian et al., 2019)
	osteoclast differentiation	m6A	Increased METTL3 expression and decreased ALKBH5 expression	Facilitating m6A modifications of MYD88‐RNA and then inducing the activation of NF‐κB	(Yu et al., 2020)
	Osteoblast activity	m6A	Increased miR‐103‐3p level and decreased METTL14 expression	Regulating the maturation process of miR‐103‐3p, which directly targets METTL14 to inhibit osteoblast activity	(Sun, Wang, et al., 2021)
	Maintenance of bone mass	m6A	Increased FTO expression	Demethylating and enhancing the stability of the mRNAs of Hspa1a and other genes that can protect osteoblasts from genotoxic damage	(Zhang et al., 2019)
	Osteoporosis	m6A	Increased FTO expression	Regulated the stability of Pparg mRNA	(Shen et al., 2018)
Fertility Decline	Ovarian aging	m6A	Decreased FTO expression	Increasing the stability of FOS mRNA	(Jiang et al., 2021; Sun, Zhang, et al., 2021)

## AUTHOR CONTRIBUTION

Z.J., D.S., L.H., Y.G., and G.L. conceived the manuscript. Z.J., D.S., L.H., S.S., and L.Z. drafted the manuscript, made the figures, and summarized the Tables. HI.L., P.G., G.V., Y.G., and G.L. finalized the manuscript, figures, and Tables. L.H. and Y.G. significantly contributed to the manuscript revision.

## CONFLICT OF INTEREST

The authors declare that they have no conflict of interest.

## Data Availability

Data sharing is not applicable to this article as no new data were created or analyzed in this study.
